# Combined Displacement and Angle Sensor with Ultra-High Compactness Based on Self-Imaging Effect of Optical Microgratings

**DOI:** 10.3390/s24030908

**Published:** 2024-01-30

**Authors:** Mengdi Zhang, Hao Yang, Qianqi Niu, Xuye Zhang, Jiaan Yang, Jiangbei Lai, Changjiang Fan, Mengwei Li, Chenguang Xin

**Affiliations:** 1 School of Instrument and Electronics, North University of China, Taiyuan 030051, Chinasz202306117@st.nuc.edu.cn (J.L.);; 2 School of Instrument and Intelligent Future Technology, North University of China, Taiyuan 030051, China

**Keywords:** self-imaging effect, optical micrograting, combined sensor, multi-degree of freedom, displacement

## Abstract

In this paper, an ultracompact combined sensor for displacement and angle-synchronous measurement is proposed based on the self-imaging effect of optical microgratings. Using a two-grating structure, linear and angular displacement can be measured by detecting the change of phase and amplitude of the optical transmission, respectively, within one single structure in the meantime. The optically transmitted properties of the two-grating structure are investigated in both theory and simulation. Simulated results indicate that optical transmission changes in a sinusoidal relationship to the input linear displacement. Meanwhile, the amplitude of the curve decreases with an input pitch angle, indicating the ability for synchronous measurement within one single compact structure. The synchronous measurement of the linear displacement and the angle is also demonstrated experimentally. The results show a resolution down to 4 nm for linear displacement measurement and a maximum sensitivity of 0.26 mV/arcsec within a range of ±1° for angle measurement. Benefiting from a simple common-path structure without using optical components, including reflectors and polarizers, the sensor shows ultra-high compactness for multiple-degrees-of-freedom measuring, indicating the great potential for this sensor in fields such as integrated mechanical positioning and semiconductor fabrication.

## 1. Introduction

Precision measurement with multiple degrees of freedom (DOF) can be used to accurately detect the position and presence of objects in planar or three-dimensional space, which have been widely used in machining positioning and motion driving in ultra-precision machining processes [1,2,3]. The measured degrees of freedom typically include linear parameters (e.g., displacement in three linear axes) and angular parameters (e.g., roll angle, yaw angle, and pitch angle) [4,5]. In the past decades, several optical methods have been reported for multiple-DOF measurement, including laser interferometry, autocollimation, and grating diffraction interferometry [6,7,8]. Among these methods, the grating-based approach shows high resolution and stability for compact machining systems [9,10]. Generally, multiple-DOF measurement based on optical gratings can be demonstrated by either using multiple linear displacement sensors or combining the optical interference and the autocollimation [11,12,13]. We considered the recent compelling need for developing ultracompact components for high-precision machining systems such as lithography machines [14]. In 2013, X. Li et al. presented a multi-axis surface encoder to measure 6-DOF translational displacement motions and angular motions of a planar motion stage. The measurement resolutions of the displacement and angle are about 1 nm and 0.1 [15]. In 2022, S. Wang et al. proposed a grating encoder that can provide absolute 4-DOF position and pose monitoring with sub-arcsecond and sub-micron accuracy [16]. In this case, plenty of optical components, including reflectors, polarizers, and wave plates, are required for synchronously measuring the linear and angular parameters, resulting in low compactness and significant degradation of accuracy caused by large Abbe errors and cross-talk errors [17,18]. 

The self-imaging effect reveals a phenomenon that, when a periodic structure (e.g., optical grating) is irradiated by a plane wave, the intensity distribution of the optical field at certain distances behind the structure shows the same period [19]. Benefiting from a simple optical path and high compactness, either linear or angular measurement has been demonstrated in past years based on the self-imaging effect of optical gratings. For linear displacement measurement, in 2014, P. Rodriguez-Montero et al. presented a device for measuring displacement based on self-imaging and non-steady photo-electromotive force effects, demonstrating an estimated resolution better than 10 μm within a dynamic range of 1.5 mm [20]. In 2015, S. Agarwal et al. reported an in-plane displacement measurement by using a circular grating Talbot interferometer [21]. By analyzing the shift of self-imaging interferometric fringe patterns, a resolution at the micrometer level was reported. In 2022, C. Xin et al. improved the resolution to 0.73 nm within a range up to mm level by using a two-quadrant detector [22]. For angular measurement, in 1999, Q. Liu et al. found the Moire fringes changing with different parallelism between two optical gratings [23]. In 2006, A. Wang et al. reported a sensor that used the self-imaging effect to detect the local intensity and incident angle of light [24]. In 2022, Z. Yang et al. reported an ultracompact angular displacement sensor using a double-grating structure with a sensitivity of 0.19 mV/arcsec within a range of ±396 arcsec [25]. Since both the linear and angular displacements are synchronously changing the amplitude of the output signal or the patterns of the self-imaging images in the cases mentioned above, the linear and angular displacements are hard to distinguish from each other. By using different parameters (e.g., amplitude and phase, respectively) of the output signals, the self-imaging effect can be in principle used in developing multiple-DOF measuring with high compactness and accuracy.

In this paper, an ultra-compact composite displacement and angle sensor based on the self-imaging effect of optical microgratings is demonstrated. By detecting the change in the phase and the amplitude of optical transmission behind two gratings, the linear displacement and angle can be measured synchronously. The simulated results obtained by a finite-difference time-domain (FDTD) method show that the transmission changes sinusoidally with a relative linear displacement between two gratings. The phase of the sinusoidal signal is related to the input linear displacement (e.g., a phase of 2π corresponding to a linear displacement equal to one single period of the grating). The amplitude of the sinusoidal signal attenuates with an increasing pitch angle for the upper grating. A resolution of 4 nm for linear displacement and 3.85 arcsec within a range of ±1° for angle measurement have been demonstrated experimentally, indicating the ability for synchronous multiple-DOF measurement. Benefiting from a simple coaxial optical path without using many optical components such as reflectors and polarizers, this sensor shows ultra-high compactness for multi-DOF measurement without significant degradation of accuracy compared to traditional one-dimension measurement [22], showing the great potential in applications ranging from integrable high-precision machining to manufacturing.

## 2. Principle

The measuring principle of the proposed sensor can be explained by a plane wave interference theory. A double-layer structure consisting of two optical microgratings with the same period is used. When a monochromatic plane wave is vertically incident on an optical grating, the amplitude transmission behind the grating can be expressed as [26] follows:

(1) $$t\left(x\right)=\sum_{n=-\infty }^{\infty }C_{n}\exp\left(i2\pi \frac{n}{d}x\right)$$

where *C_n_* is the Fourier coefficient and *d* is the grating period.

**Figure 1:**
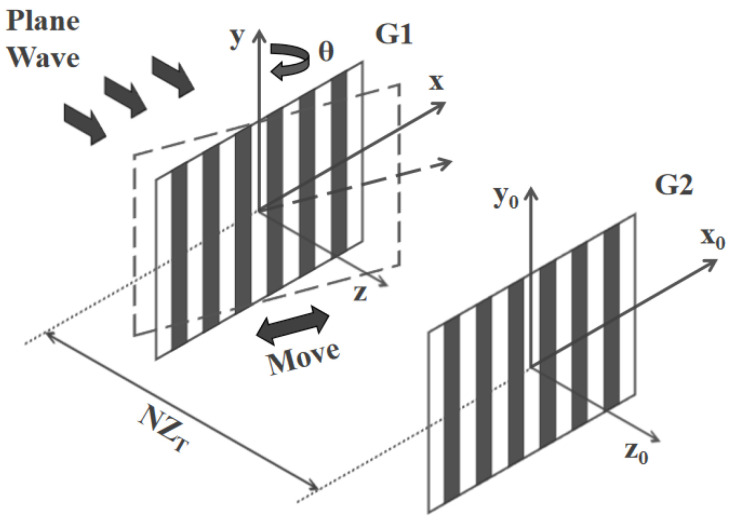
Schematic diagram of the two-grating structure. G1 is the upper grating. G2 is the lower grating. G1 twists around the *y* axis with a pitch angle of *θ* and moves along the x axis linearly.

A schematic diagram of the two-grating structure is shown in Figure 1. Assuming that G2 is located behind G1 with a certain distance of *NZ_T_* (*N* = 0, 1, 2…), where

(2) $$Z_{T}=\frac{2d^{2}}{\lambda }$$

is the period of the self-imaging images in the direction perpendicular to the plane of grating [27]. 

Assuming that G1 rotates along the *y* axis with an angle of *θ* synchronously, the two gratings are no longer parallel to each other. Using a projective simplification, G1 can be projected into a parallel plane after being rotated. In this case, the modified grating period (*d*) can be given by *d*cos*θ*, and self-imaging positions are defined by *Z* = *Z_T_′* = 2*d*^2^cos^2^*θ*/*λ*.

Assuming that the distance between G1 and G2 is *Z*, the complex amplitude distribution of the light field at the lower surface of G2 can be expressed as follows:

(3) $$U\left(x,z\right)=\exp\left(ikz\right)\sum_{n=-\infty }^{\infty }C_{n}\exp\left(i2\pi \frac{n}{d\cos\theta }x\right)$$

Assuming that G1 and G2 have a relative displacement, the complex amplitude distribution behind G2 can be given [25] as follows:

(4) $$U\prime \left(x,z,\Delta L\right)=\exp\left(ikz\right)\sum_{n=-\infty }^{\infty }\sum_{m=-\infty }^{\infty }C_{n}C_{m}\exp\left[i2\pi \frac{\left(n/\cos\theta \right)+m}{d}x\right]\exp\left[i2\pi \frac{n/\cos\theta }{d}\Delta L\right]$$

where Δ*L* is the relative linear displacement between G1 and G2. 

Equation (4) indicates that *U*′(*x*,*z*,Δ*L*) changes sinusoidally with an input linear displacement. Since the system can be regarded as a low-pass filter in which the components whose spatial frequency is higher than that of the gratings are cut off, the phase of the sinusoidal curve is related to the displacement by a factor of 1/*d*cos*θ*, which means that each change of 2π in phase corresponds to a linear displacement of *d*cos*θ* [23]. Meanwhile, the amplitude of the curve is related to the input angle by a factor of 1/cos*θ*. As a result, it is possible, in principle, to measure the linear and angular displacement at the same time by detecting the phase and the amplitude, respectively.

## 3. Simulation

**Figure 2:**
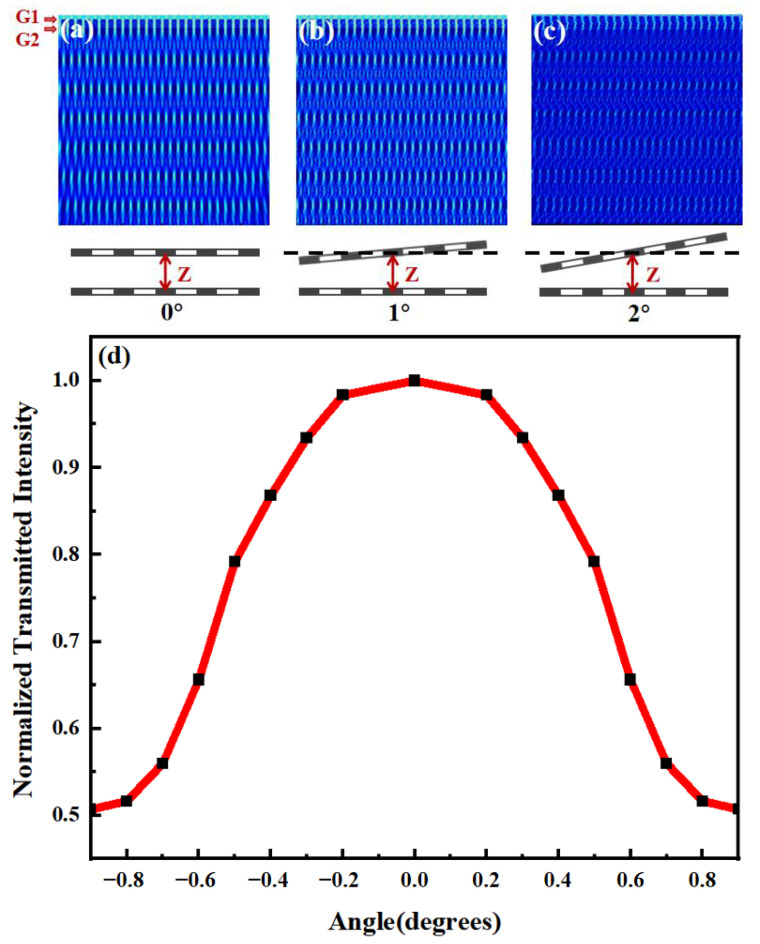
Simulated optical transmission behind two optical gratings with (**a**) *θ* = 0°, (**b**) *θ* = 1°, and (**c**) *θ* = 2°. The distance between the two gratings does not change in the simulation. The positions of the two gratings are indicated by the red arrows. The period of the gratings used in the simulation is 4 μm. (**d**) Simulated transmitted intensity with different *θ*.

The optically transmitted properties of a double-grating structure are investigated by the FDTD method. The material of the gratings is aluminum. The period of the gratings is 4 μm. The wavelength of the input beam is 635 nm. The simulated optical transmission with different *θ* is shown in Figure 2a–c. As *θ* increases from 0° to 2° gradually, the transmission decreases, agreeing with the theoretical analysis. The normalized simulated transmitted intensity with different *θ* is shown in Figure 2d. A maximum normalized intensity of 1 is obtained with *θ* = 0°. As |*θ*| increases, the normalized intensity decreases as well (e.g., down to 0.51 as |*θ*| = 0.9°).

**Figure 3:**
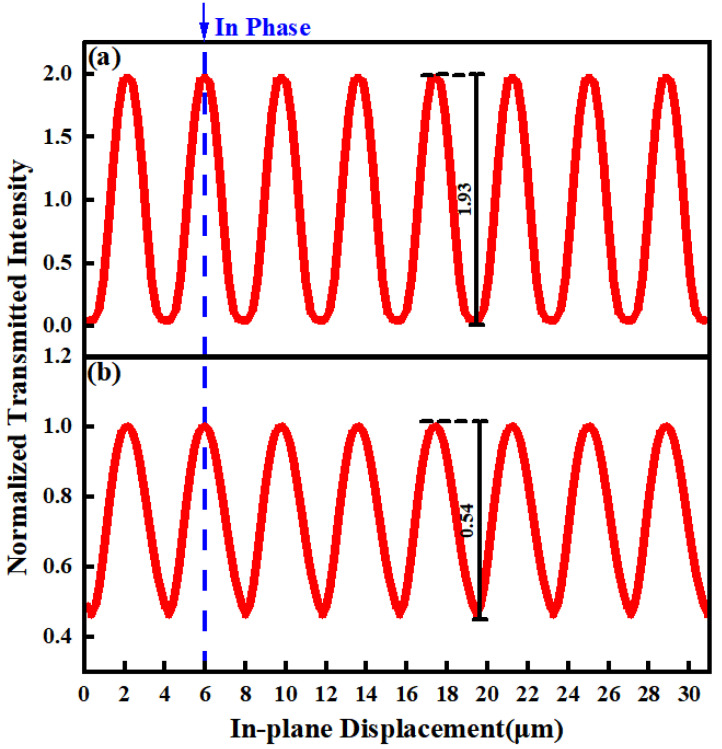
The transmitted intensity with different *θ* values of (**a**) 0° and (**b**) 1°, respectively, as there is an input linear displacement.

The transmitted intensity with different *θ* of 0° and 1°, respectively, as there is an input linear displacement, is shown in Figure 3. With a relative linear displacement between G1 and G2 along the in-plane direction perpendicular to the grating lines, the transmitted intensity changes sinusoidally with the input displacement in both cases. However, the change of *θ* results in a different amplitude. The amplitude of the sinusoidal signal decreases from 1.93 to 0.54 as *θ* changes from 0° to 1°. It is worth mentioning that, despite the different amplitudes, the sinusoidal signals in the two cases approximately remain in the same phase with a small, rotated angle, which means that the input linear displacement and angle can be distinguished by the phase and amplitude, respectively.

## 4. Experiment

### 4.1. Self-Imaging Effect of One Single Optical Grating

**Figure 4:**
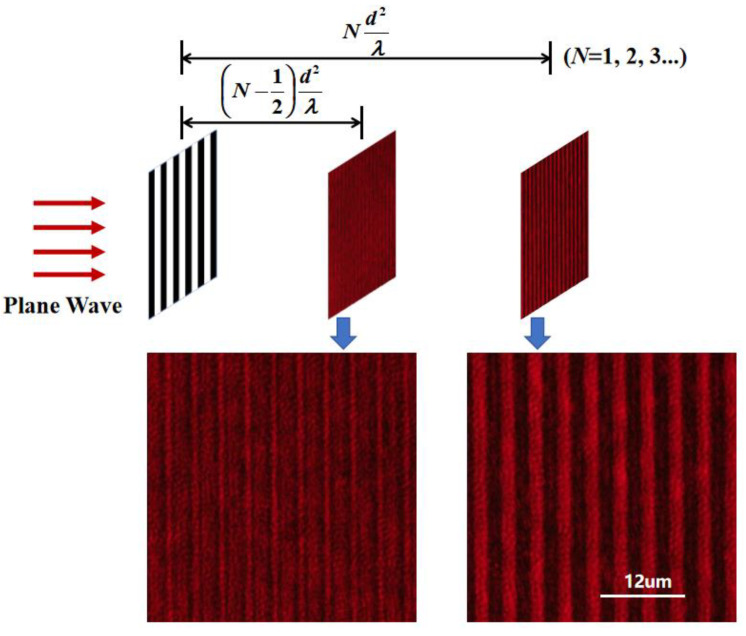
The self-imaging patterns behind a 4-micrometer-period grating at different positions obtained experimentally.

The self-imaging patterns behind a 4-micrometer-period grating are shown in Figure 4. The wavelength of the input plane wave is 635 nm. A microscope system consisting of a 40× object lens and a CCD (M830, Murzider, Dongguan, China) is located behind the grating. As the microscope system moves along the optical axis, the self-imaging patterns at different locations behind the grating can be obtained. Subdivided patterns and self-imaging patterns with periods of 2 μm and 4 μm are obtained at positions of (*N*-½)*d^2^*/*λ* and *Nd^2^*/*λ* (*N* = 1, 2, 3…), respectively, agreeing with the theoretical analysis of the self-imaging effects [28]. 

### 4.2. Combined Displacement and Angle Measurement with Double Gratings

**Figure 5:**
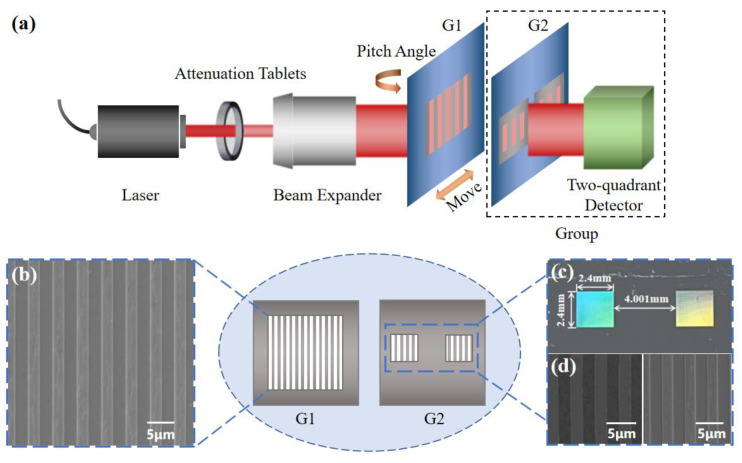

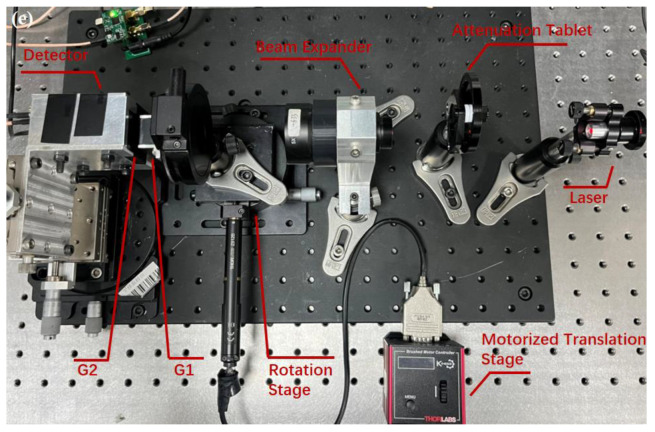
(**a**) Schematic diagram of the combined displacement and angle sensor. (**b**) Scanning electron microscope image of G1 used in the experiment. (**c**) The optical photo of G2. (**d**) Scanning electron microscope image of the two quadrants on the G2 used in the experiment. (**e**) Optical image of the experimental setup.

The schematic diagram of the experiment is shown in Figure 5. The beam with a wavelength of 635 nm from the laser (CPS635R, Thorlabs, Newton, NJ, USA) was irradiated onto two gratings. The gratings are prepared by etching aluminum film with a thickness of 150 nm, which is located on a silicon dioxide substrate with a thickness of 500 μm. The structure of the grating and the image under the electron microscope are shown in Figure 5b,d. The scanning electron microscopy image demonstrates a grating period of 4 µm and a duty ratio of 0.5. G2 has a two-quadrant structure (as shown in Figure 5c), in which the two grating quadrants are located on a single substrate with a distance of 4.001 mm [22]. The distance of 4.001 mm corresponds to a phase difference of (1000 + 1/4) × 2π, resulting in two sinusoidal signals from the two quadrants of the detector with a difference of π/2 in phase according to Equation (4). A multi-quadrant detector (OSQ100-IC, OTRON, Shanghai, China) is placed behind G2 to measure the transmitted intensity.

**Figure 6:**
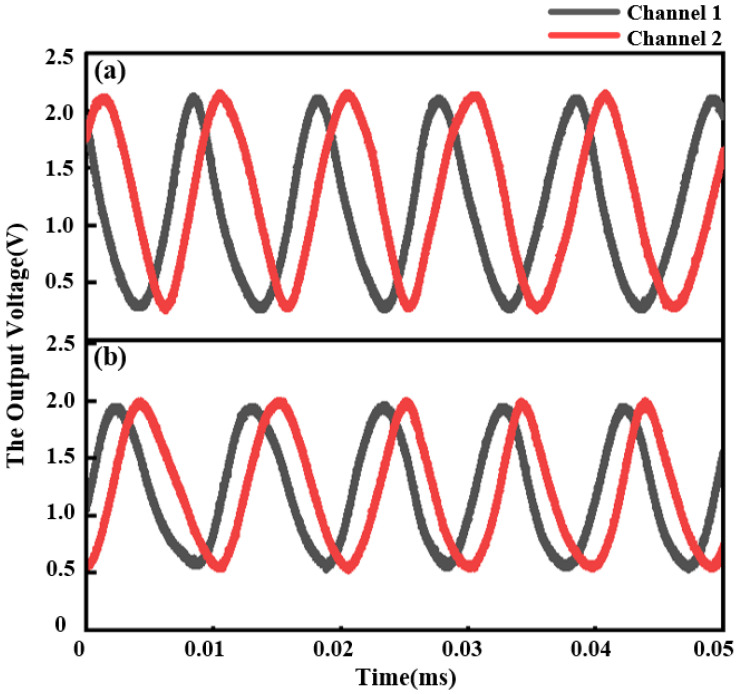
Experimental results with different *θ* of (**a**) 0° and (**b**) 1°, respectively, with a uniform-rate input linear displacement.

The sinusoidal signals obtained from the multi-quadrant detector with different *θ* are shown in Figure 6. According to the simulated results mentioned above, the amplitude of the output signals decreases from 1.88 V to 1.4 V as *θ* changes from 0° to 1°. With a uniform-rate input linear displacement, the phase of the signals changes meanwhile.

**Figure 7:**
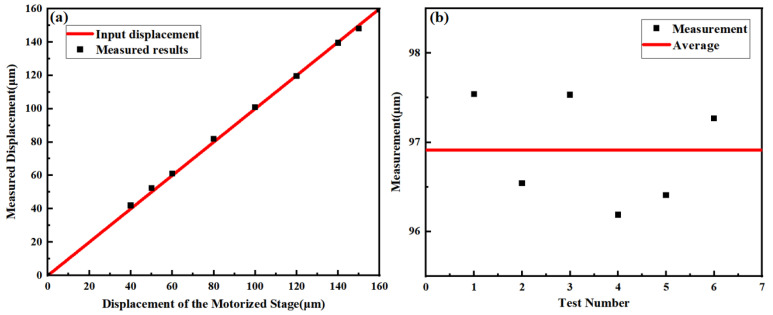
(**a**) Linear displacement measurement results obtained experimentally. The black triangle symbols indicate the results obtained from the proposed sensor. The red line indicates the input displacement. (**b**) Multiple measurement results with an input linear displacement of ~100 μm. The black dots indicate the measured results. The red line indicates the average value.

Associated with an interpolation circuit with a subdivision factor of 1000, the displacement can be measured by counting the output square signals [22]. The experimentally measured results for linear displacement within a range of 160 μm are shown in Figure 7a. A motorized translation stage (MT1/M-Z8, Thorlabs, Newton, NJ, USA) is used to provide linear displacement. The results show good agreement between the measured results and the input displacement with a maximum error of 2.4 μm, which may result from the Abbe error and the environmental vibration. Multiple measurement results shown in Figure 7b indicate an accuracy within ±1 μm. Considering the positional repeatability of ±0.7 μm for the translation stage used in the experiment, the results show high accuracy.

According to Equation (4), the resolution (*S*) of the displacement measurement can be given by [22]:

(5) $$S=\frac{d\cos\theta }{C}$$

where *C* is the subdivision factor of the interpolation circuit. As *θ* is small, the resolution of the linear displacement is calculated to be around 4 µm/1000 = 4 nm.

**Figure 8:**
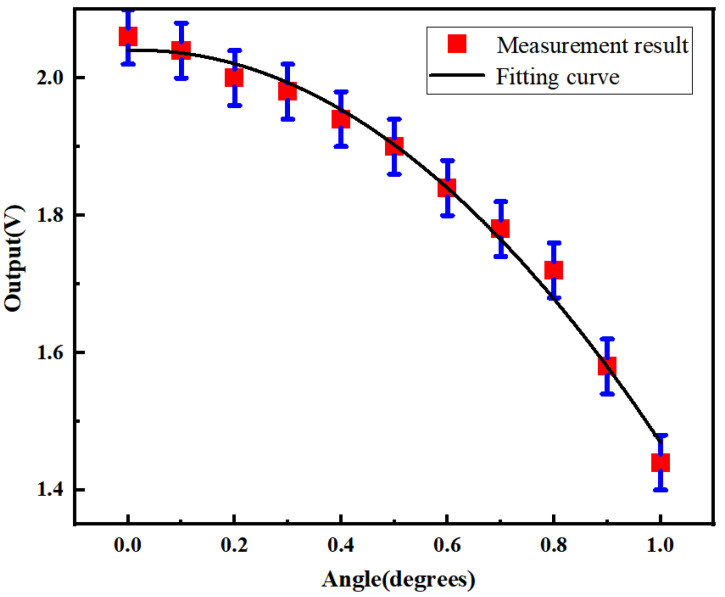
The relationship between the rotated angle of G1 and the amplitude of the output signal from the detector. The red dots indicate the measured results. The black line shows the fitting curve. The error bar is mainly caused by the positional error of the rotation stage.

The relationship between the rotated angle of G1 and the amplitude of the output signal from the detector is shown in Figure 8. The single-axis rotary table (RSM82-1A, Zolix, Beijing, China) used in the experiment enables a rotation with a resolution of 2′ within a range of 360°. The results show a downtrend, which agrees with the simulated results. A maximum slope of 0.92 V/degree is obtained by a fitting curve with a R2 of 0.98969, resulting in a maximum sensitivity of up to 0.26 mV/arcsec. Considering the resolution of the voltage detector of 1 mV for the oscilloscope (TBS2204B, Tektronix, Beaverton, QR, USA) used in the experiment, the maximum total resolution of the proposed sensor for angular measurement is calculated to be ~3.85 arcsec.

The influence of the fluctuation of the input power is also discussed. The total sensitivity (*S*) can be defined by the following:

(6) $$S=S_{1}\times S_{2}$$

where *S*_1_ and *S*_2_ are the optical sensitivity and the electrical sensitivity, respectively. *S*_1_ represents the change of transmitted power to the input angle. *S*_2_ represents the change of output voltage to transmitted power.

We measured the input power with a laser power meter (FieldMate, Coherent, Palo Alto, CA, USA) within a 30-minute time frame. The results show a measured power of 1.19 ± 0.01 mW, indicating a fluctuation within ±0.84%. As a result, *S*_1_ also changes within a small fluctuation of ±0.84%. Considering the good linear output properties of the detector used in the experiment with an input power below 1.2 mW, *S*_2_ remains almost unchanged with such a small fluctuation. According to Equation (6), the total sensitivity should, in principle, show a small error within ±0.84% as a result of the fluctuation of the input power.

## 5. Conclusions

In this paper, we propose a combined sensor for measuring displacement and angle synchronously based on the self-imaging effect of optical microgratings. Using a double-grating structure, the linear displacement and angle can be measured by detecting the change in phase and amplitude of the output sinusoidal signals, respectively. Both the simulated and experimental results show that the transmitted intensity changes sinusoidally with an input linear displacement. The amplitude of sinusoidal signals decreases with an increasing rotational angle. Associated with an interpolation circuit with a subdivision factor of 1000, linear displacement and angle measurement with a resolution of 4 nm and 3.85 arcsec, respectively, are demonstrated experimentally, which is comparable to methods such as grating diffraction interferometry and photoelectric autocollimation [29]. Since the measurement of displacement and angle is operated within one single structure, the proposed sensor shows an ultracompact structure. It is worth mentioning that, benefiting from the single common optical path, there is no significant degradation of accuracy observed in the experiment. By using interpolation circuits with a higher subdividing factor and optimizing the location of G2, a better resolution of both the linear displacement and angle measurement may be obtained. The results show the great potential of this sensor for integrated high-precision multi-DOF measurement in applications ranging from lithography machines to precision machine tools.

## Data Availability

The data presented in this study are available in the article.
